# Anti-Mullerian Hormone: Usefulness in Clinical Practice

**DOI:** 10.1155/2013/791386

**Published:** 2013-12-24

**Authors:** Kai J. Buhling, Petra Stute, Volker Ziller

**Affiliations:** ^1^Department of Gynecological Endocrinology, Clinic of Gynecology, University Hospital Hamburg-Eppendorf, Hamburg, Germany; ^2^Department of Gynecological Endocrinology and Reproductive Medicine, University Hospital Bern, Bern, Switzerland; ^3^Department of Gynecological Endocrinology and Reproductive Medicine, University Hospital Giessen and Marburg, Marburg, Germany

*Anti-Mullerian hormone* (AMH) is a protein that inhibits the development of the mullerian ducts (paramesonephric ducts) in the male embryo. AMH prevents the development of the mullerian ducts into the uterus and other mullerian structures in the first 8 weeks of gestational age. AMH is produced by granulosa cells of the ovary during the reproductive years and controls the formation of primary follicles by inhibiting excessive follicular recruitment by FSH.

Whereas AMH is nearby undetectable in females at birth, the level appears to be fairly stable in healthy young women from puberty to the age of 30 years.

Some endocrine disorders are correlated with elevated (PCOS) or lower AMH levels (premature ovarian insufficiency). Additionally some studies have shown that the dose of stimulation in assisted reproduction therapy (ART) is negatively correlated to serum AMH levels. Due to this association some authors stated that serum AMH reflects well the ovarian follicular reserve.

The aim of the special issue is to summarize the actual view and the clinical aspects of this interesting hormone in different reproductive life stages.

The colleagues N. Josso et al. and M. L. Johansen et al. give an actual statement about the use of AMH in the pediatrics endocrinology.

A. La Marca et al. have focused on the inter- and intraindividual changes and especially the impact of ethnicity and body mass index as well as smoking behaviours on the AMH levels. They also highlight the impact of hormonal suppression of the ovarian function and its influence on the AMH levels.

R. Tal and D. B Seifer give a review about the racial and ethnical differences.

One manuscript deals with the usefulness of AMH in the diagnosis of ovarian epithelial cancer. Another manuscript describes the AMH levels as a possible marker of reproductive function after different cancers in young women after chemotherapy.

Since AMH is also present in male, two manuscripts have been selected concerning this topic: the paper by E. Matuszczak et al. gives an overview about the physiology and pathology of AMH levels in male and the second one, written by R. P. Grinspon et al., deals with the AMH levels in precocious puberty treated with GnRH.

We believe that AMH and it is clinical usefulness are a topic that is of importance in scientific and clinical practice. This special issue provides important facts and adds to the actual discussion about the clinical usefulness of AMH. We also appreciate gaining some overview about the meaning of AMH in males.

*Kai J. Buhling* *Kai J. Buhling* *Petra Stute* *Petra Stute* *Volker Ziller* *Volker Ziller*

